# Fibrosis Related Inflammatory Mediators: Role of the IL-10 Cytokine Family

**DOI:** 10.1155/2015/764641

**Published:** 2015-06-24

**Authors:** Erna Sziksz, Domonkos Pap, Rita Lippai, Nóra Judit Béres, Andrea Fekete, Attila J. Szabó, Ádám Vannay

**Affiliations:** ^1^MTA-SE, Pediatrics and Nephrology Research Group, Bokay Janos Street 53-54, Budapest 1083, Hungary; ^2^1st Department of Pediatrics, Semmelweis University, Bokay Janos Street 53-54, Budapest 1083, Hungary; ^3^MTA-SE, Lendület Diabetes Research Group, Bokay Janos Street 53-54, Budapest 1083, Hungary

## Abstract

Importance of chronic fibroproliferative diseases (FDs) including pulmonary fibrosis, chronic kidney diseases, inflammatory bowel disease, and cardiovascular or liver fibrosis is rapidly increasing and they have become a major public health problem. According to some estimates about 45% of all deaths are attributed to FDs in the developed world. Independently of their etiology the common hallmark of FDs is chronic inflammation. Infiltrating immune cells, endothelial, epithelial, and other resident cells of the injured organ release an orchestra of inflammatory mediators, which stimulate the proliferation and excessive extracellular matrix (ECM) production of myofibroblasts, the effector cells of organ fibrosis. Abnormal amount of ECM disturbs the original organ architecture leading to the decline of function. Although our knowledge is rapidly expanding, we still have neither a diagnostic tool to detect nor a drug to specifically target fibrosis. Therefore, there is an urgent need for the more comprehensive understanding of the pathomechanism of fibrosis and development of novel diagnostic and therapeutic strategies. In the present review we provide an overview of the common key mediators of organ fibrosis highlighting the role of interleukin-10 (IL-10) cytokine family members (IL-10, IL-19, IL-20, IL-22, IL-24, and IL-26), which recently came into focus as tissue remodeling-related inflammatory cytokines.

## 1. Introduction

The significance of chronic fibroproliferative diseases (FDs) including pulmonary fibrosis, chronic kidney disease (CKD), inflammatory bowel diseases (IBD), and cardiovascular or liver fibrosis is rapidly increasing and they have become a major public health problem [1]. According to current estimates nearly 45% of all deaths are attributed to FDs; thus, they are the leading cause of morbidity and mortality in developed countries [2, 3].

Different FDs share common features such as chronic inflammation which shows a correlation with the progression of fibrosis. In the injured organs chemotactic stimuli trigger the rapid recruitment of immune cells including macrophages and neutrophils. These infiltrating immune cells then produce numerous proinflammatory cytokines and growth factors, which trigger the activation of myofibroblasts (MFs), the main effector cells of tissue remodeling [4]. Under physiological conditions remodeling leads to the almost complete regeneration of the tissue without permanent traces of injury. However, in the case of chronic FDs the sensitive balance between the synthesis and degradation of extracellular matrix (ECM) components is disturbed, and the continuously activated MFs produce an excessive amount of ECM resulting in the replacement of parenchymal tissue by connective tissues. This chronic pathogenic remodeling process leads finally to the destruction of normal organ architecture and consequent decline of its function [5, 6].

Despite the unmet medical need there is no generally accepted therapy to treat or hinder fibrosis. Since inflammation plays an unequivocal role in the development of fibrosis, new therapeutic strategies targeting the inflammatory pathways may offer promising opportunities. Thus, the aim of the present review is to summarize the main events of organ fibrosis with special focus on tissue remodeling-related inflammatory mediators, highlighting the potential pathomechanical role of the members of interleukin-10 (IL-10) cytokine family.

## 2. Main Cellular Events of Organ Fibrosis

Chronic inflammation, as a common hallmark of FDs, is initially represented by the recruitment of neutrophils and macrophages; however, almost all immune cell types including type 1 T helper (Th1), Th2, Th17, regulatory T (Treg) and B lymphocytes, and eosinophil and basophil granulocytes are involved in the process. These immune cells and also the injured inherent cells of the affected organ, such as endothelial and epithelial cells, release a wide range of inflammatory cytokines and growth factors [7, 8] including IL-13 or transforming growth factor- (TGF-) *β*, which contribute either to the maintenance of chronic inflammation [9] or to the proliferation and enhanced ECM production of MFs.

**Figure 1:**
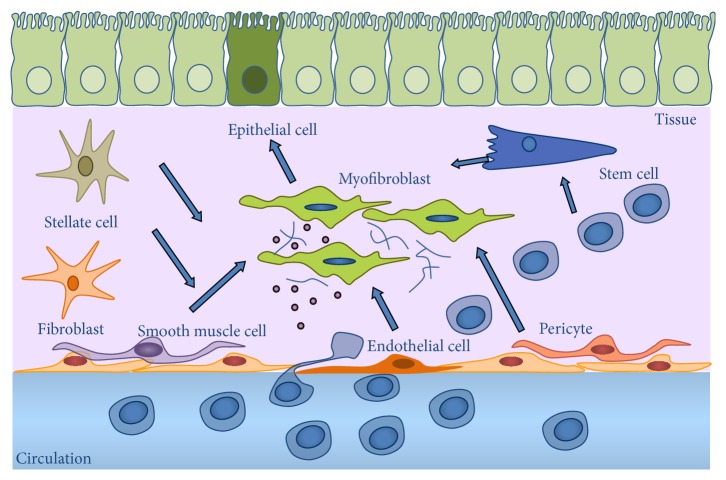
Hypothetical origin of myofibroblasts.

MFs, as the main effector cells of organ fibrosis, are *α*-smooth muscle actin (*α*-SMA) positive, spindle, or stellate-shaped cells lacking the epithelial or endothelial markers, such as cytokeratins or cluster of differentiation (CD) 31 [10, 11]. Although the origin of MFs is controversial, they may arise by the phenoconversion of different cell types including fibroblasts [12], pericytes [13], stellate [14], smooth muscle [15], epithelial [16], endothelial [17], and stem cells or circulating progenitors [18–20] (Figure 1). After their activation MFs proliferate and produce an excessive amount of different ECM components including fibrillar collagens including collagens I, III and glycoproteins such as fibronectin, fibrillin, elastin, and proteoglycans [10] and nonfibrillar collagens including collagen IV, a main component of the basal membranes [21]. However, the relative contribution of the different infiltrating and inherent cell types in the injured organ to the formation of MFs is still not clear.

## 3. Main Inflammatory Mediators of Organ Fibrosis

Chronic inflammation leads to the release of a wide range of inflammatory mediators, which can contribute either to the stimulation (profibrotic) or to the inhibition (antifibrotic) of fibrosis (Table 1). In the present section we discuss the biological role of the most well-studied mediators in the complex process of organ fibrosis. It is widely accepted that TGF-*β*1 is the central element of the “core pathway” of organ fibrosis in most if not in all organs, including the airways [22], kidney [23], gastrointestinal tract [24], heart [25], and liver [26]. TGF-*β* is mainly derived from macrophages and fibroblasts [27]; however, other immune and nonimmune cells including dendritic cells [28], Treg [29], CD8^+^ T [30], or epithelial cells [31] can also produce it. Binding of TGF-*β* to its receptor complex leads to the phosphorylation of the downstream signaling mediators small mothers against decapentaplegic homolog (SMAD)2/3 forming a complex with SMAD4 [32] that translocates from the cytoplasm into the nucleus and induces the expression of its target genes. However, TGF-*β* can also promote some noncanonical signaling pathways including the activation of extracellular signal-regulated kinase (ERK)/cJun/p38 mitogen activated protein kinases [33]. In response to the activation of these TGF-*β*-mediated signaling pathways MFs differentiate from their precursors and express *α*-SMA [34]. Other growth factors including platelet-derived growth factor (PDGF), connective tissue growth factor (CTGF), insulin-like growth factor (IGF), fibroblast growth factor (FGF), and epidermal growth factor (EGF) also influence the complex molecular interplay leading to the differentiation and increased ECM production of MFs [35–37]. These growth factors have been also implicated in the pathomechanism of a number of fibrotic diseases including lung [38, 39], kidney [40, 41], intestinal [42, 43], heart [44], and liver fibrosis [45].

Th2-derived cytokines (IL-4, IL-5, IL-6, IL-13, and IL-21) have distinct role in the regulation of organ fibrosis [4]. One of the most studied Th2 interleukins is IL-13, which exerts a strong profibrotic effect in different FDs. In animal models pulmonary overexpression of IL-13 induced subepithelial airway fibrosis [94]; its inhibition with anti-IL-13 antibody significantly reduced ECM deposition after bleomycin-induced lung fibrosis [95]. Previously it has been suggested that the biological role of IL-13 is partially due to the profibrotic effects of IL-4 [96], as they share the common IL-4R*α*/signal transducer and activator of transcription 6 (STAT6) signaling pathways [97]. However, recently it has been demonstrated that IL-13 still explicates its fibrosis-inducing effect when the canonic IL-4R*α*/STAT6-mediated signaling pathway is blocked. Indeed, IL-13 has been demonstrated to activate an additional signaling mechanism through its own receptor (IL-13R*α*2) leading to organ fibrosis [98]. Other Th2 cytokines including IL-5 and IL-21 can enhance the profibrotic effect of IL-13 by increasing its production and/or the expression of its receptor [99, 100]. However, IL-21 can promote tissue fibrosis also by inducing the differentiation of naive T cells to Th17 cells [27].

Th17 cells produce a variety of different cytokines: among them IL-17 is the most well-studied one. IL-17 was shown to contribute to the development of fibrosis in different organs including the lung [101], kidney [102], intestine [103, 104], heart [105], and liver [106]. Elevated level of IL-17 was found in human intestinal strictures and MFs expressed its receptor IL-17RC during fibrosis associated with Crohn's disease (CD). Indeed, IL-17 induces the collagen production of subepithelial MFs and the expression of matrix metalloproteinase-3 (MMP-3), MMP-12, and tissue inhibitor of MMPs (TIMP)-1 in the colon, which have significant effects on the ECM remodeling and tissue architecture [103, 104]. Pharmacologic inhibition of the IL-17-induced ERK1/2 or p38 signaling pathways attenuated the collagen expression of mouse hepatic stellate cells [106, 107]. The profibrotic effect of IL-17 was suggested in relation to skin fibrotic lesions as well. IL-17 gene knockout (KO) mice had diminished bleomycin-induced skin fibrosis and IL-17 deficiency attenuated skin thickness in a mouse model of scleroderma [108].

## 4. Characteristic of the IL-10 Cytokine Family

In addition to the above described growth factors and cytokines, recently members of the IL-10 family as a new group of fibrosis-related inflammatory mediators came into focus. Cytokines of the IL-10 family exert host defense mechanism; they are essential for the maintenance of epithelial layer integrity and also facilitate tissue healing. IL-10 cytokine family consists of nine related molecules: IL-10, IL-19, IL-20, IL-22, IL-24, IL-26, IL-28A, IL-28B, and IL-29 [109]. These cytokines can be classified into three subfamilies with different biological functions: (1) IL-10 subfamily represented by IL-10 itself; (2) IL-20 subfamily (IL-19, IL-20, IL-22, IL-24, and IL-26) which play a role in host defense mechanisms against bacteria; (3) type III interferons (IFNs): IL-28A, IL-28B, and IL-29, which induce antiviral responses.

Initially, IL-10 was described as a Th2 cytokine but later it has been revealed that many other immune cells including Th1, Th17, Treg, CD8^+^ T, and B lymphocytes, macrophages, dendritic, natural killer, and mast cells also express IL-10 [109, 110]. Binding of IL-10 dimers to their tetramer receptor consisting of two IL-10R*α* and two IL-10R*β* chains activates tyrosine kinase 2 and Janus tyrosine kinase 1 (JAK1), which phosphorylate IL-10R*α*. Then STAT3 binds to IL-10R*α* and gets phosphorylated by JAK1. Finally phosphorylated STAT3 translocates into the nucleus and binds to the STAT-binding elements in the promoters of various IL-10 target genes. One of these IL-10 responsive genes is the suppressor of cytokine signaling 3 (SOCS3), whose induction was correlated with decreased expression of TNF-*α* and IL-1*β*. Moreover, IL-10 can affect the expression of other downstream effectors including MMP-9, inducible nitric oxide synthase, and IFN-*γ* [109, 111]. IL-10 also inhibits the activation of antigen presenting cells through reducing the expression of major histocompatibility complex class II. IL-10 has a general suppressive effect; it inhibits both the innate and adaptive immune responses, thus preventing increased exacerbations. Thereby IL-10 plays a significant role in the prevention of tissue damage which is a common element of chronic FDs. Indeed, wound repair results in scar formation in IL-10 KO mice [112] and on the contrary overexpression of IL-10 modulates inflammatory responses at a wound site of adults more closely resembling the profile characteristic for the embryo [113]. These observations suggest that by reducing the inflammatory response IL-10 may inhibit the proliferation and collagen synthesis of the MFs as well [114].

Based on their overlapping target-cell profile and biological function, IL-19, IL-20, IL-22, IL-24, and IL-26 were classified into the IL-20 subfamily [115]. Cellular sources of the members of IL-20 subfamily are immune cells including monocytes, lymphocytes, natural killer cells, and macrophages and also epithelial cells and fibroblasts. Members of the IL-20 subfamily show significant similarities in the structure of their receptor heterodimers. While IL-19 binds to IL-20RA/IL-20RB, IL-20 and IL-24 bind either to IL-20RA/IL-20RB or to IL-22RA1/IL-20RB heterodimers. IL-22 binds to IL-22RA1/IL-10RB and IL-26 to the IL-20RA/IL-10RB receptor complex. Expression of the different receptor heterodimers can vary between tissues, which may explain the organ-specific effects of the members of this cytokine subfamily. IL-10RB is ubiquitously expressed in the haematopoietic system, IL-20RA and IL-20RB are primarily distributed in the skin, lung, testis, ovary, and placenta, and IL-22RA1 was shown to be present in the kidneys, intestine, liver, pancreas, and skin [115]. Similar to IL-10, binding of the members of IL-20 subfamily to their receptors activates the JAK1/STAT1 and STAT3 signaling pathways [115]. IL-22 and IL-24 both can act also through the Akt, ERK, JNK, and p38 signaling pathways [92, 116].

Finally, the third subgroup of IL-10 family is the subfamily of type III IFNs (IL-28A, IL-28B, and IL-29) which signal through the IFN-*λ* receptor (IFN*λ*R). IFN*λ*R is a heterodimer consisting of an IL-28R*α* and an IL-10R*β* subunit and is present exclusively on the surface of epithelial cells. Ligand binding to IFN*λ*R induces the activation of JAK/STAT signaling and antiviral activity on epithelial surfaces [117]. Unlike the other members of the IL-10 family type III IFNs have no known effect on organ fibrosis.

## 5. Role of IL-10 Family Members in Fibrotic Diseases

### 5.1. Pulmonary Fibrosis

Interstitial lung disease (ILD) is a heterogenic group of disorders with different etiology. ILD can be linked to a certain environmental exposure, including cigarette smoking, chemotherapy or radiation therapy, infection, or autoimmune diseases; however, it can also appear without any known cause. In this case, it is termed as idiopathic pulmonary fibrosis (IPF) [118]. Most of these pulmonary disorders are primarily characterized by inflammation of the lung interstitium [119]. However, others such as IPF are primarily fibrotic and are associated with excessive deposition of ECM resulting in the disruption of the original architecture of the lung and loss of its volume. In general, patients with a known etiology of ILD respond well to the targeted therapy especially when inflammation dominates; however, they are difficult to treat when fibrosis comes into view [119]. Indeed treatment opportunities for IPF are limited; lung transplantation is the only therapeutic option available in severe cases.

Recent studies reveal close association between IL-10 family of cytokines and ILDs. Level of IL-10 was significantly increased in the lung and bronchoalveolar lavage (BAL) of silica inhaled mice compared to controls. Moreover, IL-10 KO mice had more increased lung inflammation after intratracheal instillation of silica than wild type animals [47]. Moreover, genetic delivery of IL-10 significantly attenuates the TGF-*β* production in the lung of mice with bleomycin-induced pulmonary fibrosis [48]. In humans greater percentage of peripheral CD4^+^ T lymphocytes produced IL-10 and higher serum levels of IL-10 were found in patients with IPF than normal subjects [49]. Moreover, the extent of IL-10 production correlated with forced vital capacity of the patients [120]. Similarly, comparing the protein concentration of IL-10 in the bronchoalveolar lavage (BAL) of patients with different types of ILDs the highest level of IL-10 was demonstrated in patients with IPF compared with sarcoidosis or hypersensitivity pneumonitis [50–52].

Similar to IL-10 the protective role of IL-22 is suggested in relation to fibrotic lung disorders. Indeed, it has been demonstrated that recombinant IL-22 treatment inhibits the phenoconversion of alveolar epithelial cells into MFs, thus reducing the number of ECM producing cells in a bleomycin-induced mouse model of lung fibrosis [78]. Administration of an anti-IL-22 neutralizing antibody has also been shown to enhance pulmonary inflammation and ECM deposition in the same bleomycin-induced model of lung fibrosis.

Similar results were found in a mouse model of hypersensitive pneumonitis induced by repeated exposure to* Bacillus subtilis* leading to lung fibrosis. Namely, inhibition of IL-22 resulted in enhanced collagen deposition in the lung, whereas treatment with recombinant IL-22 inhibited lung fibrosis [79]. These beneficial antifibrotic effects of IL-22 suggest its potential as a novel therapeutic target in the treatment of pulmonary fibrosis.

To the best of our knowledge there are no studies investigating the role of IL-19, IL-24, IL-26, IL-28, and IL-29 in pulmonary fibrosis.

### 5.2. Renal Fibrosis

The prevalence of CKDs is estimated to be 8–16% worldwide and their number is rapidly increasing [121]. Currently, about 20–25 million patients need renal replacement therapy [122]. The most common etiologies of CKDs and renal fibrosis are diabetes mellitus (DM) and hypertension in the adult population [122, 123] and obstructive nephropathy in childhood [124]. However, CKDs irrespectively of their etiology always have an inflammatory component, which shows a strong correlation with the progression of fibrosis and the decline of renal function [125–127].

Recently, the connection between IL-10 and renal fibrosis has been suggested. Jin et al. demonstrated that after the onset of unilateral ureteral obstruction (UUO) more severe inflammation and fibrosis develop in the kidney of mice lacking IL-10 than in wild type controls. Following UUO they found increased infiltration of inflammatory cells and upregulation of inflammatory chemokines and cytokines including monocyte chemoattractant protein-1, RANTES, tumor necrosis factor- (TNF-) *α*, IL-6, IL-8, or macrophage colony-stimulating factor in the kidney of IL-10 knockout (KO) mice. In line with increased inflammation in the mice lacking IL-10 they found a more pronounced collagen I deposition and increased expression of fibronectin, *α*-SMA, fibroblast-specific protein-1, vimentin, and MMP-2 supporting the development of renal fibrosis [53]. In accordance with the observation of Jin et al., Rodell et al. demonstrated that local immunotherapy with IL-10 in hyaluronic acid hydrogels reduces macrophage infiltration, the number of apoptotic cells, and the size of the fibrotic area as well, confirming the potential use of IL-10 containing hydrogels in the local treatment of CKD [54, 55].

The participation of other members of the IL-10 family like IL-19 or IL-20 in the pathomechanism of renal fibrosis is less unequivocal. Elevated urinary level of IL-19 [70] and IL-20 [74] was observed in patients with CKD.* In vitro* treatment of human renal proximal tubular epithelial cells with nephrotoxic agents, including Adefovir, Dipivoxil, Cisplatin, or Ifosfamide, was shown to induce the expression of IL-19 [70]. Similarly, HgCl_2_ treatment of HK-2 human proximal tubular epithelial cell line resulted in increased presence of IL-20 and its receptors [75]. Moreover, administration of either IL-19 [71] or IL-20 [75] induced the apoptosis of renal tubular epithelial cells* in vitro*.

A recent study demonstrated that following renal ischaemia-reperfusion (I/R) injury the serum level of IL-22 and also the expression of its receptor, IL-22R1, in the renal proximal tubular epithelial cells are increased [80]. Treatment of the animals with recombinant IL-22 or the overexpression of IL-22 decreased the I/R-induced tubulointerstitial injury in the cortex and outer medulla and also the serum urea and creatinine levels compared to saline-treated control animals. The underlying mechanism of the beneficial effects of IL-22 is its overall antiapoptotic effect. Indeed overexpression of IL-22 upregulated the renal expression of B-cell lymphoma-2 (Bcl-2) and downregulated that of Bcl-2-associated death promoter in mice subjected to I/R injury [80]. However, hypoxia is a known inducer of organ fibrosis; to the best of our knowledge, there is no data in the literature directly supporting the role of IL-22 in the pathomechanism of renal fibrosis. The involvement of other members of the IL-10 cytokine family like IL-24, IL-26, IL-28, and IL-29 in renal fibrosis is completely unknown.

### 5.3. Intestinal Fibrosis

Intestinal fibrosis is a serious complication of IBD in both adults and children [128–131] and more than 60% of patients with IBD require one or more operations over their lifetime, commonly because of stricture formation [128, 132]. However anti-inflammatory therapies reduce the symptoms of IBD, the recently available treatments of intestinal fibrosis are insufficient, and new therapeutic approaches are needed.

Similar to other chronic diseases experimental and clinical studies suggest the involvement of the members of IL-10 family in intestinal fibrosis. The first study reporting elevated level of IL-10 in the serum of patients with both active CD and ulcerative colitis (UC) was published in 1995. Also increased expression of IL-10 was found in the mucosa of patients with UC in remission [56]. Later studies, however, do not confirm these results unequivocally. Indeed normal levels of IL-10 in patients with IBD [57, 58] and lower expression of its receptors IL-10R1 and IL-10R2 in patients with remission were also revealed [76]. Moreover, loss of function mutations in the gene of IL-10 or its receptor causes early onset of IBD with refractory colitis and perianal disease [59]. In line with these findings decreased production of IL-10 was observed in whole blood cell cultures of patients with severe phenotypes, compared with nonpenetrating, nonstricturing CD patients. Similarly, DCs isolated from patients suffering from penetrating CD produced less IL-10 in response to lipopolysaccharide (LPS) stimulation compared to patients without complications [133]. These observations suggest that defects in the production of the anti-inflammatory IL-10 may represent a mechanism mediating the more severe manifestations of CD. Despite the apparent discrepancy in the literature regarding the expression of IL-10 in patients with IBD, the treatment with IL-10 or IL-10-inducing agents could be of particular benefit, because IL-10 itself can suppress proinflammatory responses with a consequential limitation of tissue damage and may exert antifibrotic effects as well. Recently clinical trials are in progress investigating the effect of the supplementation of IL-10 in IBD (see more details later in the therapy section of this review) [60, 61].

Similar to IL-10, the protective role of IL-19 was also suggested in IBD. Indeed, IL-19 KO mice were more susceptible to DSS-induced experimental colitis than the wild type animals. The lack of IL-19 in the IL-19 KO mice correlated with the accumulation of macrophages. Moreover, macrophages derived from IL-19 KO mice produced significantly higher level of inflammatory cytokines including IL-6, TNF-*α*, and IL-12 following LPS stimulation compared to macrophages of wild type animals [73]. In humans decreased production of IL-19 was observed in the monocytes and peripheral blood mononuclear cells (PBMCs) of patients with active CD compared to those from healthy controls. Moreover, administration of recombinant IL-19 significantly decreased the production of TNF-*α* in LPS-treated monocytes and PBMCs of healthy controls but not in those of the patients with active CD [72].

Previous studies demonstrated that production of IL-20 can be induced by LPS, TNF-*α*, and other proinflammatory cytokines [76, 115]. The number of epithelial and inflammatory cells expressing IL-20 and IL-20RB was increased in the mucosa of patients with active UC, but level of IL-20 was decreased in the colonic mucosa of patients with UC in remission compared with patients with active UC and controls [76].

Elevated serum and mucosal level of IL-22 was demonstrated in patients with active CD that correlated with disease severity [81, 131, 134, 135]. IL-22 is a direct downstream effector cytokine of IL-23, whose receptor was identified by the Genome Wide Association Study as an IBD-related gene [136]. Elevated level of IL-23 was found in patients with active IBD [137] and blocking of IL-23 was effective in both prevention and treatment of active colitis [138], suggesting the potential of the IL-23-IL-22 pathway as a target of further therapeutic interventions. Contrary to these findings in human IBD, studies on mouse models of colitis suggested the protective role of IL-22 in the intestine. Inhibition of IL-22 by neutralizing antibodies in wild type mice or the lack of IL-22 in KO mice with dextran-sodium-sulphate- (DSS-) induced colitis resulted in an increased inflammation and epithelial damage of the colon leading to more severe weight loss of the animals [82, 83].

Moreover, rIL-22 treatment of colonic epithelial cells isolated from mice with DSS-induced colitis induced activation of STAT3 signaling pathway that regulates gut homeostasis and was shown to promote wound healing in an IL-22-dependent manner [82].

The expression of IL-24 was shown to be significantly elevated in the mucosa of patients with active CD and UC compared to that of inactive IBD or controls, but the number of IL-24-producing peripheral B, CD4^+^ T, CD8^+^ T cells and monocytes was increased only in patients with active CD but not in UC patients or controls [91]. Andoh et al. investigating the effect of IL-24 treatment on the behaviour of HT-29 colonic epithelial cells found that IL-24 activates the JAK1/STAT3 and also the SOCS3 signaling pathway and leads to increased expression of membrane-bound mucin-1, mucin-3, and mucin-4 supporting its suppressive effects on mucosal inflammation in IBD [92].

Level of IL-26 was also elevated in the serum and inflamed region of the colonic mucosa of patients with CD and it was expressed by infiltrating immune cells mainly by Th1 and Th17 but not epithelial cells [93]. IL-26 can promote the expression of proinflammatory cytokines through the activation of STAT1/3, ERK1/2, JNK, and Akt signaling pathways suggesting its proinflammatory role in IBD [139].

To the best of our knowledge there are no data about the direct role of IL-10 family in the pathomechanism of intestinal fibrosis; however, the above-mentioned data suggest their relevance in aberrant intestinal tissue remodeling. The involvement of IL-28A and IL28-B and IL-29 in IBD is still completely unknown.

### 5.4. Cardiac Fibrosis

Cardiac fibrosis is a common feature of different pathological conditions including myocardial infarction, pressure overload, hypertrophic cardiomyopathy, viral infections, toxic insults, or metabolic disturbances [63, 140, 141]. Recently a series of animal studies suggested the protective effect of IL-10 in cardiac fibrosis. An* in vivo* experiment using IL-10 KO and wild type mice suggested that lack of IL-10 results in adverse tissue remodeling and more severe myocardial fibrosis in an isoproterenol-induced pressure overload-derived heart failure model. On the other hand, administration of recombinant IL-10 improved cardiac remodeling and inhibited scar tissue formation and reduced the mortality of mice [62, 63].

The further* in vivo* studies confirmed the role of IL-10 in tissue scaring using other animal models as well. In ischemia-induced mouse model of heart fibrosis impaired mobilization of bone marrow-derived endothelial progenitor cells, which are crucial in neovascularization and tissue repair, was observed in the heart of IL-10 KO mice compared to wild type controls. Moreover, IL-10 treatment of the mice enhanced the survival of the endothelial progenitor cells leading to better myocardial recovery [142]. Similarly, IL-10 treatment of the mice suffering from autoimmune myocarditis resulted in a significant decrease of myocardial inflammation and fibrosis. Furthermore, the administration of IL-10 prevented the relapse of the left ventricular function and increased the ejection fraction [65].

The development of chronic cardiomyopathy in the experimental* Trypanosoma cruzi*-infected dog model of Chagas disease was strongly correlated with the production of IL-10. Indeed low level of IL-10 and simultaneously high expression of IFN-*γ* and TNF-*α* were observed in the acute cardiac infection phase, which correlated with the severity of heart inflammation and fibrosis in the chronic phase [64].

However, our knowledge is limited, and elevated level of IL-22 was demonstrated in the heart of mice with dilated cardiomyopathy and cardiac fibrosis. Treatment of mice with IL-22-specific antibody decreased the survival rate of the animals and exacerbated myocardial fibrosis suggesting the cardioprotective role of IL-22 through the inhibition of myocardial fibrosis [84].

The role of other members of the IL-10 family including IL-19, IL-20, IL-24, IL-26, IL-28, and IL-29 in cardiac fibrosis is completely unknown.

### 5.5. Liver Fibrosis

Liver fibrosis is one of the major causes of morbidity and mortality worldwide with around 1.5 million deaths per year [143]; however, the exact pathomechanism is just partially understood. The main causes of liver fibrosis include fatty liver, alcohol abuse, biliary track disease, chronic viral infection, autoimmune disease, and toxicant exposure [144].

The most studied members of the IL-10 family related to liver fibrosis are IL-10, IL-20, and IL-22. Studies investigated the involvement of these cytokines mainly in alcoholic hepatitis, nonalcoholic and infection associated liver fibrosis [145].

In the liver IL-10 can be produced by a variety of cell types including hepatocytes, Kupffer cells, sinusoidal endothelial cells, hepatic stellate cells, and lymphocytes and also its receptor is expressed by progenitor and hepatic stellate cells, the predominant cell types involved in liver fibrogenesis [66, 85].

Higher hepatic TNF-*α* levels and more severe liver fibrosis can be observed in the carbon tetrachloride (CCl_4_) treated IL-10 KO mice than in the wild type animals [67, 68].

On the contrary, IL-10 gene therapy reduced the expression of profibrotic genes, including TGF-*β* and TNF-*α*, and reversed the thioacetamide-induced hepatic fibrosis in mice [69]. Recent studies demonstrated that IL-10 plays a protective role in alcoholic liver disease as well [146].

Similar to IL-10, elevated level of IL-20 was found in hepatocytes and hepatic stellate cells of patients suffering from liver fibrosis. However, contrary to the effect of IL-10, recombinant IL-20 treatment of mice enhanced the expression of the profibrotic cytokines including TGF-*β* and TNF-*α* and promoted the collagen synthesis of the liver. Treatment with neutralizing antibody against IL-20 or IL20RA diminishes the CCl_4_-induced liver fibrosis in mice. Also IL-20 KO mice are less sensitive against CCl_4_-induced liver fibrosis [77].

A variety of studies reveal the relevance of IL-22 in liver fibrosis demonstrating its protective role. In a chronic-binge ethanol feeding mouse model of alcohol induced liver injury the recombinant IL-22 treatment of the animals ameliorated liver injury and alcoholic fatty liver through the activation of STAT3 signaling pathway [85, 86]. In the same murine model of hepatic fibrosis administration of IL-22 upregulated the expression of several antiapoptotic and antioxidant genes contributing to the attenuation of the oxidative stress [86]. Long-term administration of recombinant IL-22 to mice with a high fat diet induced hepatic steatosis and diminished the TNF-*α* level of the liver [87].

Overexpression of IL-22 or recombinant IL-22 treatment decreased the expression of alpha-smooth muscle actin (*α*SMA) in cultured hepatic stellate cells and also in the fibrotic liver of the mice with CCl_4_-induced liver fibrosis. In addition, IL-22 promoted the senescence of hepatic stellate cells through the SOCS3 bound p53 and p21 signaling pathways, thereby ameliorating liver fibrosis [88].

On the contrary, inhibition of IL-22 with a neutralizing antibody reduced the activation of STAT3 and led to the worsening of liver injury in a T cell-mediated hepatitis induced by concanavalin A [89].

In humans, elevated level of IL-22 was found in the serum and liver tissue of human patients with HCV-induced and alcoholic liver fibrosis. Based on the results of Wu et al. profibrotic effects of IL-22 were proposed in humans in contrast with its antifibrotic role in mice suggesting that IL-22 may have diverse functions in different species and disease states [90].

The role of other members of the IL-10 family including IL-19, IL-24, IL-26, IL-28, and IL-29 needs to be elucidated in liver fibrosis.

## 6. Therapeutic Targets of Tissue Remodeling

**Figure 2:**
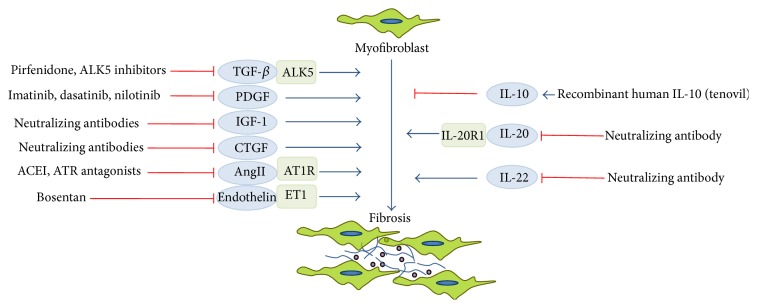
Potential therapeutic targets of fibrosis. ACEI: angiotensin-converting enzyme inhibitors; ALK5: activin-linked kinase 5; Ang II: angiotensin II; ATR: angiotensin receptor; CTGF: connective tissue growth factor; ET1: endothelin receptor isoform 1; IGF-1: insulin-like growth factor-1; IL: interleukin; PDGF: platelet-derived growth factor; TGF-*β*: transforming growth factor-*β*.

Chronic FDs affect hundreds of millions of people and are the leading cause of morbidity and mortality in the Western world. Despite the urgent medical need there is no generally accepted strategy to treat or hinder organ fibrosis. However, due to the efforts during the last few years there was a remarkable achievement in the treatment of organ fibrosis. The drugs which are currently under development target the key participants of the “fibrosis pathway” including TGF-*β*, PDGF, IGF, CTGF, angiotensin II, or endothelin-1 (Figure 2).

Among them, pirfenidone, targeting the TGF-*β* pathway, was recently approved by the U.S. Food and Drug Administration (FDA) for the treatment of IPF. In phase III clinical trial pirfenidone successfully reduced the progression of IPF and was associated with fewer deaths [147]. However, TGF-*β* and other key factors of organ fibrosis play also a crucial role in other significant biological processes, like embryogenesis [148], regulation of immune responses [149], or cancer development [150]. Therefore, cautions must be taken in case of organ fibrosis, which is often related to chronic diseases when the antifibrotic treatment needs to be maintained for years. Therefore, besides the inhibition of the above-mentioned determinative pathways it seems to be preferable to alter new, more fibrosis-specific or endogenously antifibrotic pathways leading to fewer serious side effects and allowing life-long treatment of the patients.

Recently, the members of the IL-10 family came into focus as possible new target molecules, which may alter the progression of organ fibrosis (Table 2). Different therapeutic strategies were developed to influence the effects of IL-10, IL-20, IL-22, or IL-20RA. Among them, investigations aiming at the alteration of the IL-10 mediated pathways are in the most advanced stage. Indeed, after the successful preclinical experiments, clinical studies using human recombinant IL-10 (rhuIL-10) are already in progress for the treatment of IBD. A double-blind clinical trial enrolling patients with CD after intestinal resection demonstrated that administration of rhuIL-10 for 12 consecutive weeks was safe and well tolerated [61]. Another double-blind placebo controlled trial with 95 active CD patients who received rhuIL-10 (sc.; 1, 5, 10, or 20 *μ*g/kg/day) for 29 days showed that 5 *μ*g/kg/day rhuIL-10 treatment can induce clinical remission and endoscopic improvement in 23.5% of the patients compared to placebo-treated group where no remission was detectable. At the 20-week follow-up period the relapses requiring further therapeutic intervention were decreased by 15% in CD patients who were treated with 5 *μ*g/kg rhuIL-10 compared to controls treated with placebo only [151]. However, interestingly the higher than 5 *μ*g/kg/day doses of rhuIL-10 treatment were less effective [152]. CD patients treated with high doses (10 or 20 *μ*g/kg/day) of rhuIL-10 had increased production of IFN-*γ* in whole blood cells and elevated serum neopterin levels, which may be responsible for the effectiveness of higher rhuIL-10 doses in CD. Moreover, high doses of rhuIL-10 caused headache, fever, and anaemia [152].

Schreiber et al. examined 329 patients with therapy refractory CD and observed a clinical improvement in 46% of patients treated with 8 *μ*g/kg/day rhuIL-10 compared with the 27% of placebo-treated control patients; however, they did not find any significant differences in the clinical remission between rhuIL-10 (1, 4, 8, and 20 *μ*g/kg) and placebo-treated groups [153]. Marlow et al. suggested that IL-10 can be rather effective in the prevention of IBD; however, there are several individual differences between the exact etiologies of the disease. Moreover, IL-10 may exert also an immunostimulatory effect, which may compensate its immunosuppressive qualities [60].

Local treatment with IL-10-secreting* Lactococcus lactis* (*L. lactis*) prevented the development of colitis in IL-10 KO mice and reduced inflammation in the DSS-induced mouse model of colitis without systemic side effects [154]. In a human phase I trial ten CD patients were treated with a hIL-10 sequence containing* L. lactis* twice a day for one week. The treatment was safe and reduced the disease activity without any side effects observed in the case of high systemic doses [155]. Similar to* L. lactis* treatment, replication-deficient adenoviral vectors directly delivered to gastrointestinal epithelial cells were also effective in murines through the improvement of local IL-10 release [156, 157].

The above-mentioned data suggest that systemic administration of rhuIL-10 may be a safe and well-tolerated treatment contributing to the clinical improvement of CD and the local IL-10 therapy may have even more potential because of having fewer side effects. However, the direct effect of rhuIL-10 on intestinal fibrosis that often appears in IBD had not been studied in humans.

To the best of our knowledge, recently there are no human studies investigating the effect of recombinant IL-10 and other family members on lung fibrosis. In rats, inhaled IL-10 was shown to attenuate LPS-induced pulmonary and systemic inflammation through the reduction of proinflammatory mediators including TNF-*α*, IL-1*β*, IL-6, and IFN-*γ* in the BAL and plasma [158]. Moreover, after bilateral femoral fracture that induces systemic inflammation and impairs the lung function, inhalative administration of 50 *μ*g/kg/day recombinant mouse IL-10 decreased the pulmonary infiltration of neutrophils and reduced the expression of the adhesion molecule ICAM-1 but had no significant effects on the systemic inflammatory response [159].

In a double-blind, placebo-controlled study the effect of rhuIL-10 was investigated in human patients with renal transplantation who received OKT3, a monoclonal murine antibody targeting the epsilon chain of the CD3-T cell receptor complex that efficiently reverses graft rejection, as induction therapy. Wissing et al. found that pretreatment with doses of up to 1 *μ*g/kg rhuIL-10 was safe and significantly reduced the OKT3-induced release of TNF-*α* [160]. Moreover, short-term treatment of nephritic rats with intravenous (iv) rhuIL-10 was effective in the inhibition of matrix deposition and reduced the protein level of *α*-SMA in antithymocyte 1 induced glomerulosclerosis but had no beneficial effects on proteinuria [161].

In a mouse model of myocardial infarction subcutan (sc) administration of recombinant IL-10 suppressed the expression of proinflammatory cytokines in the myocardium, reduced infarct size, attenuated infarct wall thinning, improved left ventricular functions, reduced the activity of MMP-9, and diminished fibrosis [63].

In a randomized, double-blind trial twenty-four patients with chronic hepatitis C were sc. treated with either 4 or 8 *μ*g/kg rhuIL-10 per day for 90 days. The therapy was safe and well tolerated, and administration of rhuIL-10 normalized the serum levels of alanine aminotransferase (ALT), improved liver histology, and reduced liver fibrosis [162]. Long-term (12-month) rhuIL-10 sc. treatment of thirty patients with chronic hepatitis C-induced advanced fibrosis, who had failed antiviral therapy, resulted in a significant improvement of their serum ALT, decreased hepatic inflammation, and fibrosis. However, long-term administration of rhuIL-10 altered the cytokine profile of PBMCs promoting a Th2 dominance and decreased the number of HCV-specific CD4^+^ and CD8^+^ T cells resulting in enhanced HCV viral burden due to the alterations in the host's immunologic viral surveillance [163].

Moreover, in preclinical experiments the effect of treatment with recombinant IL-22 [88], anti-IL-20, or anti-IL-20RA monoclonal antibody [77] was demonstrated to inhibit TGF-*β* production or the excessive accumulation of ECM components in mouse models of liver fibrosis induced by chemical agents (CCl_4_) or mechanical bile duct ligation.

However, results about the possible use of rhuIL-10 as an antifibrotic drug are promising and further preclinical and clinical studies are needed to elucidate the precise role of the IL-10 family in fibrosis and to estimate their potential therapeutic effectiveness.

## Figures and Tables

**Table 1:** Inflammatory mediators with pro- and/or antifibrotic effects [27, 46].

	Profibrotic	Antifibrotic
Growth factors	TGF-*β* PDGF CTGF IGF FGF EGF VEGF	HGF

Cytokines	IL-1 IL-4 IL-5 IL-6 IL-13 IL-17 IL-19 IL-20 IL-21 IL-24 IL-33 TNF-*α* CCL2 CCL3 CCL4 CCL20	IL-7 IL-10 IL-12 IL-22 IFN-*γ*

TGF: tumor growth factor; PDGF: platelet-derived growth factor; CTGF: connective tissue growth factor; IGF: insulin-like growth factor; FGF: fibroblast growth factor; EGF: epidermal growth factor; VEGF: vascular endothelial growth factor; HGF: hepatocyte growth factor; IL: interleukin; TNF: tumor necrosis factor; CCL: chemokine (C-C motif) ligand; IFN: interferon.

**Table 2:** Involvement of the members of IL-10 cytokine family in pulmonary, renal, intestinal, heart, and liver fibrosis.

IL-10 family member	Lung	Kidney	Intestine	Heart	Liver
IL-10	IL-10 KO (mouse): inflammation ↑ [47] BLEO-induced pulmonary fibrosis (mouse) + IL-10: TGF-*β* ↓ [48] IPF (human): serum IL-10 ↑ [49] ILD (human): BAL IL-10 ↑ [50–52]	UUO IL-10 KO (mouse): fibrosis ↑ and inflammatory chemokines ↑ [53] UUO (mouse) + IL-10 in hyaluronic acid hydrogels: fibrosis ↓ [54, 55]	IBD (human): serum, mucosa IL-10 ↑/→ [56–58] IL-10 loss of function mutation: IBD ↑ [59] IBD + IL-10: dose dependent improvement of disease activity [60, 61]	Isoproterenol-induced fibrosis IL-10 KO (mouse): fibrosis ↑ [62] Isoproterenol-induced fibrosis (mouse) + IL-10: fibrosis ↓ [63] Myocarditis (dog): serum IL-10 ↓ and fibrosis ↑ [64] Autoimmune myocarditis + IL-10 (mouse): fibrosis ↓ [65]	Acute, chronic liver injury (mouse): IL-10 ↑ [66] IL-10 KO + CCl_4_: fibrosis ↑ [67, 68] Thioacetamide-induced liver fibrosis + IL-10 gene therapy (mouse): fibrosis ↓ [69]

IL-19	—	CKD (human): urinary IL-19 ↑ [70] Nephrotoxic agents (epithelial cells): IL-19 ↑ [70] Renal tubular epithelial cells + IL-19: apoptosis ↑ [71]	IBD (human): serum IL-19 ↓ [72] IL-19 KO (mouse): fibrosis ↑ [73]	—	—

IL-20	—	CKD (human): urinary IL-20 ↑ [74] Nephrotoxic agents (renal epithelial cells): IL-20 ↑ [75] Renal tubular epithelial cells + IL-20: apoptosis ↑ [75]	Active IBD (human): serum, mucosa IL-20 ↑ [76]	—	Liver fibrosis (human): IL-20 ↑ [77] CCl_4_-induced liver fibrosis (mouse): IL-20 ↑ [77] CCl_4_-induced liver fibrosis + IL-20 neutralizing sb/IL-20 KO: fibrosis ↓ [77]

IL-22	BLEO-induced pulmonary fibrosis (mouse) + IL-22: fibrosis ↓ [78] BLEO-induced pulmonary fibrosis (mouse) + IL-22 neutralizing ab: fibrosis ↑ [78] HP (mouse) + IL22: fibrosis ↓ [79] HP (mouse) + IL22 neutralizing ab: fibrosis ↑ [79]	I/R (mouse): serum IL-22 ↑ [80] I/R (mouse) + IL-22: tubulointerstitial injury ↓ [80]	IBD (human) serum, mucosa IL-22 ↑ [81] DSS colitis (mouse) + IL-22 neutralizing ab: inflammation ↑ [82] DSS colitis IL-22 KO (mouse): inflammation ↑ [83]	Dilated cardiomyopathy, cardiac fibrosis (mouse): IL-22 ↑ [84] Cardiac fibrosis (mouse) + IL-22 neutralizing ab: fibrosis ↓ [84]	Ethanol-induced liver fibrosis (mouse) + IL-22: liver injury ↓, antiapoptotic, antioxidant genes ↑ [85, 86] High fat diet (mouse) + IL-22: liver TNF-*α* ↓ [87] CCl_4_-induced liver fibrosis (mouse) + IL-22: fibrosis ↓ [88] Concanavalin-induced hepatitis + IL-22 neutralizing ab: liver injury ↑ [89] HCV and alcoholic liver fibrosis (human): serum, liver IL-22 ↑ [90]

IL-24	—	—	Active IBD (human): serum IL-24 ↑ [91] HT-29 + IL-24: membrane-bound mucin-1, mucin-3, and mucin-4 ↑ [92]	—	—

IL-26	—	—	IBD (human): serum IL-26 ↑ [93]	—	—

IL-28	—	—	—	—	—

IL-29	—	—	—	—	—

ab: antibody; BAL: bronchoalveolar lavage; BLEO: bleomycin; CCl4: carbon tetrachloride; CKD: chronic kidney disease; DSS: dextran-sodium-sulphate; HCV: hepatitis C virus; HP: hypersensitive pneumonitis; HT-29: colonic epithelial cell line; I/R: ischaemia-reperfusion; IBD: inflammatory bowel disease; IL: interleukin; ILD: interstitial lung disease; IPF: idiopathic pulmonary fibrosis; KO: knockout; r: recombinant; TGF: tumor growth factor; TNF: tumor necrosis factor; UUO: ureteral obstruction.
